# A gendered magnifying glass on COVID-19

**DOI:** 10.1186/s12948-020-00129-2

**Published:** 2020-08-04

**Authors:** Lorenzo Salvati, Benedetta Biagioni, Emanuele Vivarelli, Paola Parronchi

**Affiliations:** 1 Department of Experimental and Clinical Medicine, University of Florence, Largo Brambilla 3, 50134 Florence, Italy; 2 SOD Immunologia e Terapie Cellulari, AOU Careggi, Florence, Italy; 3 Allergy Unit, AOU A. Meyer, Florence, Italy; 4 SOD Immunoallergologia, AOU Careggi, Florence, Italy

**Keywords:** COVID-19, SARS-CoV-2, Gender, Sex, Female, X chromosome, IL-6, TLR7

## Abstract

COVID-19 pandemia is affecting Countries worldwide with a gendered death excess as being a male represents, especially in the 50–69 years age group, an unfavourable factor. Females are constitutionally prone to defend themselves against pathogens with a stronger efficiency than males. As a fact, several genes involved into the regulation of the innate and adaptive immune response are strategically placed on the X-chromosome and, among them, pathogen-related receptors (PRRs), such as Toll-like receptor 7, suitable to recognize ssRNAs and trigger a gendered successful anti-viral fight. On the other hand, a more regulated IL-6 production and a more contained inflammation after the encounter of a pathogen supply score points in favour of the female sex in the view that an abnormal and exaggerated cytokine release does represent the hallmark of the deathful SARS-CoV-2 infection. The sex-prevalent expression of the attachment and permissive molecules ACE2 and TMPRSS2 further supports the concept of a male-oriented vulnerability. In this review, the possible role of biological and immunological sex differences into the higher morbidity and mortality of SARS-CoV-2 between females and males are discussed.

## Background

COVID-19, the illness caused by SARS-CoV-2, is a new emerging infectious disease. Epidemiology is showing how COVID-19 is not a gender-neutral disease, but data on the possible biologic mechanisms underlying differences between males and females in terms of disease severity are still scarce. Historically, gender has been a neglected variable in infectious disease research, but it needs to be considered [1]. Deciphering gender differences in COVID-19 offers a window into the principles of immunity against SARS-CoV-2 infection.

## Main text

### The worldwide gender dimensions of COVID-19

When comparing disease incidence, almost equal distribution is observed among men and women at different ages according to the World Health Organization (WHO) case-based surveillance system as of April 18th, 2020 [2]. The male to female ratio among confirmed cases is 1.03:1 with a median age of 51 (interquartile range, IQR: 36–65) years [2]. For males, the median age is 52 (IQR 37–65) years, and for females 50 (IQR 35–64) years [2]. The ratio slightly modifies through the age groups: in the 0–9 year group the ratio is 1.16:1, in the 60–69 year it is 1.27:1, while the largest sex ratio is in the 70–79 year age group (1.34:1). More cases among females are unexpectedly found in the 20–29 year (0.85:1) age group and, accordingly to the largest number of old-aged females into the general population, into 80 years and over (0.78:1) [2]. The distribution by age and sex of confirmed COVID-19 cases reported by the WHO case-based surveillance system modifies when excluding USA, Germany and Italy: the overall male to female ratio changes to 0.95:1, while in the 0–9, 60–69 and 70–79 year age groups men always prevail [2]. In a population-based study from Iceland, fewer females than males received positive results both in the targeted testing group (11.0% vs. 16.7%, OR 1.66) and in the population screening group (0.6% vs. 0.9%, OR 1.55) [3].

Interestingly, the International Organization for gender equality and health equity Global Health 50/50 reports sex-disaggregated data [4], not always easily available from Official Health organisms. Percentages of cases in the two sexes are highly overlapping among the different Countries with the exception of India and Pakistan (cases 76% vs 24% and 77% vs 23%, respectively), also suggesting that social biases need to be carefully disclosed. However, and more importantly, from data collected so far in COVID-19, not only the rates of disease, but also mortality/morbidity and case fatality rates (CFR) need to be stratified by age and, additionally, by sex [5] (Table 1). In a recent paper from Chinese authors [6] for first assessing severity and mortality in the two sexes, the number of deceased men was almost double than women (2.4 fold) independently of age, even though no difference in susceptibility was observed. Data should be disaggregated by sex even in clinical trials when comparing the efficacy of different treatments in patients with COVID-19 [7].

**Table 1:** The gender dimensions of COVID-19

Country	SARS-CoV-2 infections			Deaths		
	Overall	Males (%)	Females (%)	Overall (N°)	Males (%)	Females (%)
Italy	230,778^a^	46	54	31,678^a^	59	41
Spain	258,760^b^	45	55	20,585^b^	57	43
Germany	172,239^c^	48	52	7723^c^	56	44
France	142,903^d^	na	na	27,834^c^	59	41
USA	1,837,803^e^	na	na	106,876^e^	56^f^	44
China	84,614^e^	51	49	4645^e^	64^g^	36
India	115,942^e^	na	na	6642^h^	73^i^	27
Pakistan	93,983^j^	74	26	11,935^j^	74	26

The sex-disaggregated data among different countries

*na* not available from official sources

^a^ISS updated May 26, 2020

^b^Ministerio de Sanidad, Actualización nº 120. Enfermedad por el coronavirus (COVID-19). updated May 29, 2020

^c^Coronavirus Disease 2019 (COVID-19) Daily Situation Report of the Robert Koch Institute, updated May 14, 2020

^d^Public Health France (SpF) https://www.santepubliquefrance.fr/maladies-et traumatismes/maladies-et-infections-respiratoires/infection-a-coronavirus/documents/bulletin-national/covid-19-point-epidemiologique, updated May 18, 2020

^e^Coronavirus disease 2019 (COVID-19) Situation Report – 137 WHO, updated June 5, 2020

^f^Provisional Death Counts for Coronavirus Disease (COVID-19), Centre of Disease Control and Prevention, CDC https://www.cdc.gov/nchs/nvss/vsrr/COVID19/index.htm

^g^National Health Commission of the PRC (http://en.nhc.gov.cn/search.html?searchText=covid+deaths)

^h^Indian Ministry of Health and Family Welfare https://www.mohfw.gov.in/index.php (2020, 04/27)

^i^https://www.firstpost.com/health/73-of-those-who-have-died-of-covid-19-are-men-health ministry-data-8233921.html (2020, 04/06)

^j^http://covid.gov.pk/

### The gender dimensions of COVID-19 in Italy: the ICU-disparity and comorbidity-equality

As of June 3rd 2020, with 233,515 confirmed cases [8] Italy was the sixth Country in the world after USA, Brazil, Russia, United Kingdom, and Spain in patient numbers. According to the COVID-19 Italian Integrated Surveillance [9], at June 3rd 2020, the male/female cases ratio was approximately 1 (men 45.9%, women 54.1%) in Italy. Hospitalizations prevailed in males in all the age groups [10] and, accordingly, a more severe clinical course was observed. Interestingly enough, the same results also emerged from the analysis of the Italian Workers’ Compensation Authority, the National Institute for Insurance against Accidents at Work [11]. At April 21st 2020, 28,381 accidents at work were reported and represented by 71.1% females and 28.9% males. Fatal cases however displayed an inverse picture with 20.4% women and 79.6% men, most grouped within 50–64 years old (68.4%) [11]. As a fact, more men required intensive care than women in all the age groups including the older patients [10]. Regarding the age distribution of the total deaths, at June 3rd 2020, almost 85% were confined after 69 years (70–79 year age group 26.8%, 80–89 years age group 40.9%, older than 90 years 17.4%) [9]. When considering the absolute number of deaths by age group, women dying for SARS-CoV-2 infection had an older age than men (median age 85 vs 79 years) [10]. As a confirm of this National panorama, in a large cohort of more than 1500 patients of the Lombardy Region admitted to ICUs, men represented more than 80% of the patients just after 40 years of age [12]. In Florence, the COCORA multidisciplinary group found that hospitalized patients were prevalently males (65.5% vs 34.5%, p 0.045) and a strikingly majority were ICU-transferred (87.5% vs 12.5%) [13]. Thus, within Italy, severe clinical course and deaths from COVID-19 are mainly observed among older, male patients confirming lower rates of severe disease among women and younger individuals overlapping data initially described in China [14]. This disparity, however, is not observed in comorbidities. Their quality, quantity and association are equally distributed and do not differ between the two sexes [15]. As a fact, among deceased patients, cardiovascular diseases including hypertension were the most common comorbidities both in men and in women and the median number of pre-existing chronic pathologies was 3 in women as well as in men [10]. This also suggests that co-existing pathologies, although representing risk factors for severe course, cannot fully explain the observed sex difference in COVID-19.

### Social versus biological risk factors in outbreaks

A careful gender analysis related to social attitudes should be always considered when disparities between men and women are observed into pandemics as of note biases. As an example, during the 2014–2016 West African outbreak of Ebola virus disease, about the two-thirds killed by the infection were women given their traditional role as caregivers and front-line health-care workers in addition to ritual local behaviours [16]. Besides the gendered risk exposure, risk perception and handling are different between sexes [17]. Gendered ideology and practice have been recently revised with the finding of an inherent globally different behaviour response to epidemic and pandemic respiratory infectious diseases [18]. Although this meta-analysis of 85 papers prevalently covers the Anglo-Saxon world with a more limited contribution from Countries highly involved into the ongoing pandemia (Italy, China, Spain), it concludes that women more likely adopt or practice non-pharmaceutical behaviors (e.g., hand washing, sanitation, quarantine compliance, mask wearing etc.) whereas, at the same time, men are only marginally more likely to adopt and practice virtuous pharmaceutical behaviors even when available (e.g., vaccinations). Further, gender discrepancies are more evident in Islamic Countries (e.g. Saudi Arabia, Pakistan, Afghanistan, Oman, Yemen) because of differential access to healthcare facilities. As an example, almost three quarters of cases in Pakistan are among men (78%) whereas women account for less than one quarter (22%) [4].

### A gendered approach to the coronavirus infections

To approach sex differences in COVID-19, common infectious respiratory diseases as community acquired pneumonia (CAP) and severe acute respiratory syndrome (SARS) may represent interesting examples to dissect whether life expectancy depends on sex differences in risky behaviours, quality of care, individual clinical characteristics, or on the biological response to pathogens. Generally speaking, men are more susceptible to infections and prone to die when infections occur [19]. In a large multicentre cohort of old adults in the USA, survival to CAP was less likely in men than in women but demographics, health behaviour, chronic health conditions, and quality of care could not explain the difference. Viceversa, a substantial different biologic response was observed between the two sexes. Higher levels of pro-inflammatory cytokines such as tumor necrosis factor (TNF)-α and Interleukin (IL)-6 mirrored by higher IL-10, and altered fibrinolysis with increased D-dimer and lower concentrations of circulating antithrombin III and Factor IX were detected in men [20]. As already reported in other infections, patterns of inflammation and pro-coagulation associated with worse outcomes.

Closer to the argument, SARS-CoV infection as responsible of the 2002–2003 pandemic SARS, actually represents a further example of sex disparity. Data from both Hong Kong and Taiwan found that male was much more severely affected than female sex. In the paper of Kalberg et al. CFR difference between the two sexes reached *p* < 0.0001 [21] and gender-related immunity was included as a possible explanation together with other possible confounding factors such as definition of the disease, different treatment regimens, smoking history or work-environment. In a comment to this latter paper [22], gender discrepancy in mortality did not diminish through the all age groups including the elders. In a preliminary paper from Chinese researchers collecting data from SARS and recent COVID-19 experience, a gender role in mortality was observed confirming male as more serious than female cases (*p *= 0.035) and numbers of men died from COVID-19 2.4 times above women with a significant p value (*p* = 0.016) [6].

Despite a more limited number of patients from the Middle East respiratory syndrome (MERS)-CoV during the 2012–2013 outbreaks in Arabia and 2015 in the Far East, sex equally matters. The excess of male cases could be undoubtedly attributed to shepherd job with camels as possible virus reservoirs in the Middle East and Gulf Countries [23], but was certainly not-depending by exposure elsewhere, such as South Korea [24]. Likewise, however, 66.7% case fatalities were sustained by men [25].

### Genes, immune regulation and environment

It is becoming more and more evident that SARS-CoV-2 strongly deals with the immune system with the consequence, in some individuals, of a dysfunctional immune response with exaggerated inflammation, immune-impairment and auto-aggression leading to disease progression [26]. In other auto-aggressive immune-mediated diseases an interactive trinity composed by genes, immune regulation and environment has been invoked as possible pathomechanism (reviewed in [27]) with innate/adaptive immunity, hormones and sex chromosomes as different players. In COVID-19 as well as SARS, a sexual dimorphism into expression and regulation of the attack and entry molecules might be a further contributor to the observed disparity between sexes.

### ACE2: a Trojan horse for SARS-CoVs

**Fig. 1:**
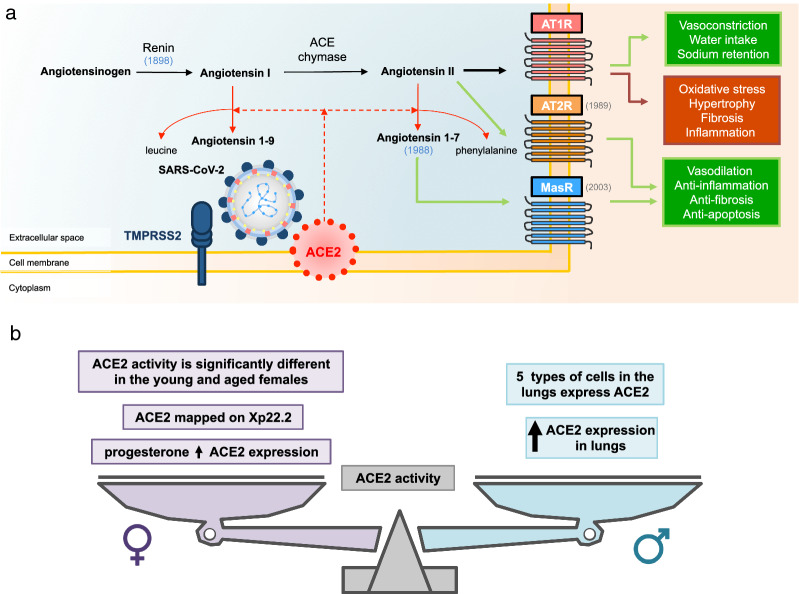
ACE2 and COVID-19. **a** The renin angiotensin system (RAS) and the role of ACE2 in SARS-CoV-2 infection in brackets the year of discovery; **b** the different balance of ACE2 expression in males and females. ACE2: Angiotensin I Converting Enzyme 2; AT_1_R angiotensin receptor type 1, AT_2_R angiotensin receptor type 2

Infection of the host target cells by Coronaviruses strictly depends on an extracellular anchor represented by a cell-surface zinc peptidase, angiotensin-converting enzyme 2 (ACE2) which is engaged by the viral spike (S) protein [28]. However, ACE2 does not represent a fully competent entry receptor as demonstrated for human pathogenic SARS-CoV and SARS-CoV-2 as well as HCoV-NL63, inasmuch as the cleavage at the S1/S2 and the S2′ site operated by host proteases is crucial to allow viral-cellular membrane fusion [29] (Fig. 1a). The viral spike protein is indeed a key determinant for transmissibility and the few changes into the aminoacidic sequences between human and animal Coronaviruses are responsible for its thousand times tightly binding to the human receptor [28].

ACE2 is a transmembrane monocarboxypeptidase with an extracellular catalytic domain operating into the RAS (renin angiotensin system). Here, its activity is to cleave away a phenylalanine from angiotensin (ANG) I thus producing the nonapeptide ANG-(1–9) and a leucine from angiotensin II producing the heptapeptide ANG-(1–7) (alamandine) with a counteracting role in respect of ANG II [30, 31]. RAS indeed operates through three different 7-transmembrane receptors differently affecting the cardiovascular and respiratory system. ANG II type 1 receptor (AT_1_R) was the first discovered as the principal receptor of ANG II and physiologically promoting vasoconstriction, water intake, and sodium retention. In pathophysiological conditions it is also responsible of nitric oxide synthesis, oxidative stress, hypertrophy, fibrosis, and inflammation. The other two receptors, ANG II type 2 receptor (AT_2_R) discovered in 1989 and the G protein-coupled receptor Mas discovered in 2003, are engaged by ANG II and ANG-(1–7), respectively. They interfere with proinflammatory pathways, resulting in vasodilation, anti-inflammation, anti-fibrosis, and anti-apoptosis and thus operate a homeostatic function in the cardiovascular system and beyond [32, 33] (Fig. 1a).

ACE2 is widely distributed in the cardiovascular system, kidneys, lungs, gastro-intestinal tract, and brain but preferring epithelial surfaces. In the lungs, ACE2 is found in type 2 pneumocytes, alveolar macrophages and ciliated bronchial epithelial cells whereas is absent on type 1 pneumocytes. Interestingly enough, type 2 pneumocytes are surfactant producers and can give raise to gas-exchange involved type 1 pneumocytes when these latter are seriously damaged such as in diffuse alveolar damage, an usual picture in SARS [34]. As a prove, viral antigens of SARS-CoV-2 have been found into the bronchial epithelial cells, macrophages and alveolar epithelia of human ACE2 transgenic mice [35]. Expression in the upper airways is more variable as present on the epithelial cells of larynx, nasal mucosa and respiratory sinuses, in tonsils and in oral mucosa, but uncertainly in trachea, vocal folds and epiglottis cells. The entire gastrointestinal tract expresses ACE2 (oesophagus, stomach, ileum and colon). In the heart, ACE2 is found on cardiomyocytes, cardiofibroblasts, and additionally into the coronary vasculature and other blood vessels on endothelial cells, myocytes, and smooth muscle cells [36]. This systemic expression, eventually co-ordinately associated with the serine-proteases necessary for virus entry, can easily explain the different clinical target symptoms observed in COVID-19.

ACE2 is tightly regulated by the TNF-α converting enzyme (ADAM-17) and apelin, among the others [30], and its expression varies over the time. Quite surprisingly, however, especially in the view of its homeostatic functions, cellular damage down-regulates ACE2 expression and no rescue system seems to operate with the effect of damage amplification and increased inflammation. In the lungs, ACE2 down-regulation associates with enhanced vascular permeability, increased lung oedema, neutrophil accumulation and worsened lung function [34]. First, several experimental models where RAS is involved, support the idea of a protective role of ACE2. As an example, in the ischemia–reperfusion kidney injury model, ACE2 knock-out compared to WT mice exhibit similar histologic injury but increased organ infiltration sustained by neutrophils, macrophages, and T cells, augmented mRNA expression of pro-inflammatory cytokines and chemokines, such as IL-1β, IL-6 and TNF-α, macrophage inflammatory protein 2 and monocyte chemoattractant protein 1 with greater apoptosis and oxidative stress [37]. Further, in viral induced lung injury ACE2 was able to protect animals [38]. Along with this, down-regulation of ACE2 has been also postulated in the human acute respiratory distress syndrome (ARDS) of ever origin and ACE2 polymorphisms associated with mortality in ARDS cohorts [39, 40]. Secondly, in the context of SARS-CoV, in vivo experimental infection as well as treatment with a Fc-fused S protein resulted in a significant reduction of ACE2 expression in the lungs paralleled by severe injury and respiratory dysfunction. Moreover, the soluble S protein was able to reduce endogenous ACE2 expression in several cell lines of respiratory and intestinal origin [41]. Finally, ACE2 shedding seems to depend on the Coronavirus strength as is highly induced by SARS-CoV and mildly by the less pathogenic HCoV-NL63 [42].

In this scenario, ACE2 might represent the first variable to justify different effects of the infection between genders (Fig. 1b).

### The sexual dimorphism of ACE2

ACE2 gene is located on the X chromosome (Xp22.2), in the Barr zone. As females carry two copies (one paternal and one maternal) of the X chromosome, with the possible result of a redoubled number of X-linked genes, this possible unbalance is compensated by a random transcriptionally silencing of one of the two X, known as X-chromosome inactivation (XCI). However, almost 15–23% of X-codified genes can escape, fully or partially, from XCI and this prerogative is proper of those genes placed in the so-called pseudoautosomal regions PAR1 and 2 [43]. The ACE2 gene is in fact placed within PAR1; however, the impact may not necessarily be a redoubled expression of ACE2 in females. By a single-cell RNA-Seq analysis performed in normal lung tissues from 6 females and 2 males [44], it was indeed confirmed ACE2 expression by type 2 pneumocytes and noticed a high expression profile heterogeneity among the tested individuals with the highest levels in an Asian male of middle age (about fivefold). In addition, almost in Chinese population, the homozygous mutation rate of SNP rs2285666 (G8790A) was much higher in males (0.550) than females (0.310) [45]. The small size of the first cohort and the limitation to Chinese donors in the second one does not allow to draw any further conclusions but it is attempting to speculate that sex (and ethnic) distinctions into viral susceptibility may exist. Deleterious variants would not be differently expressed into the Italian population to explain the observed higher lethality from SARS-CoV-2 than Chinese, however this observation comes from a still single paper and possible difference in terms of sex were not addressed [46]. Other rare ACE2 variants related to susceptibility/protection to SARS-CoV-2 were described but no relation to any particular population group or any discernible gender distribution differences were noticed [47]. Animals might indeed provide insights about a gendered ACE2 but these results could not be necessarily translated into humans. Secondly, activity and/or expression of the enzyme are addressed but they may not necessarily overlap. Third, as RAS receptors are widely distributed in the body, results from the different organs may not be equally representative of lungs. All these items may explain the variability of the observations. In rats, ACE2 expression declined with age in both sexes but was significantly higher in old female rats than male counterpart [48]. In kidneys of sheep, ACE2 mRNA and protein increase with age in both sexes but activity increases with age only in males [49]. In humans, serum ACE2 enzymatic activity recognized no sex difference but, within the women group, it was higher in the elders [50]. Finally, hormones might be an additional variable. The protective effect of oestrogens has been invoked and dissected in human cardiovascular diseases as modulating RAS components and females prevalently tip towards the ACE2/Ang(1–7)/MasR and AT_2_R pathways [51]. ACE2 protein expression and activity are indeed decreased by ovariectomy in hypertensive rats depending on the tissue [52]. In mice, however, oestrogens do not significantly interact with ERα in the lung for ACE2 receptor gene regulation [53]. Although no direct effect of progesterone has been described, ACE2 mRNA and activity are increased in the kidney and uterus of pregnant animals and ACE2 is expressed on chorionic villi [54]. Whether endogenous oestradiol or progesterone might prevent severe outcome in SARS-CoV-2 infection regulating the expression or the activity of host receptor(s) as acting as foe (attach molecule) or friend (protective anti-inflammatory), respectively, also in the lungs, it is thus still an intriguing task.

### TMPRSS2: is there any role for gender?

TMPRSS2 is a 70 kDa membrane-anchored enzyme (type 2 transmembrane serine protease) expressed on the epithelial cells of prostate where it was first described in 1997 [55]. Here, its activity is necessary to normal prostate specific antigen expression and ejaculate, even if TMPRSS2-depleted mice by serine protease domain disruption do not exhibit any alteration in survival, sexual development or reproductive impairment. TMPRSS2 is codified by a gene mapped on chromosome 21q22.3 and several androgen receptor elements are located upstream of the transcription start site and the first intron [56]. As a fact, androgens strongly upregulate TMPRSS2 expression in prostate tumor cells and they can also control, and upregulate, the oncogenic transcription factor ERG, normally androgen-insensible, when the *TMPRSS2:ERG* fusion gene is produced because of somatic gene rearrangements in prostate cancers [57].

It is no coincidence that the murine homologue of TMPRSS2 is known as epitheliasin as the enzyme is broadly expressed in other epithelia, in particular on the lining epithelia of the aerodigestive tracts (lungs and colon), in kidneys, liver, and pancreas, in addition to several cancer cells including lung cancer cell lines A549 and Calu-3, and colorectal adenocarcinoma Caco-2 [58]. However, the most interesting finding related to Coronaviruses resides on the co-expression of TMPRSS2 together with ACE2. Both molecules are found on the majority of type 2 pneumocytes as well as alveolar macrophages and epithelial cells of intrapulmonary bronchi. Here, some interstitial macrophages/dendritic cells of intra-alveolar septa of the lung can express ACE2 separately from TMPRSS2 that is present on adjacent type 2 pneumocytes. The two molecules are concomitantly present in the upper airways (buccal mucosa, nasal mucosa and respiratory sinuses, tonsils, larynx and bronchi), the gastro-intestinal tract (oesophagus, stomach, ileum and colon), the cardiovascular system (cardiac and blood vessel myocytes, endothelial cells) thus explaining the broad clinical expressions of Coronaviruses infection [36].

The cleavage activity operated by TMPRSS2 is the essential pre-requisite for viral infectivity as able to induce virus-cell membrane fusion and virus entry [29]. Other host proteases such as endosomal cathepsins concur to the priming, but they are not dispensable. The proteolytic enzyme activates S protein by splitting S1 from S2 of highly pathogenic human Coronaviruses (SARS-CoV, MERS-CoV, SARS-CoV-2) [29] but also HA1 and HA2 (Hemoagglutinin) from 2013 Asian H7N9 and H1N1 subtypes of influenza A viruses [58]. Of note, infection and death would preferentially target males in both these latter pandemics [59], although alternative data were reported from some Western Countries [60]. A genetic predisposition to a severe course from A(H1N1)pdm09 influenza infection was also found into the GG genotype rs2070788 variant carrying individuals from a Chinese cohort and a single-nucleotide polymorphism correlated with differential (and augmented) TMPRSS2 expression [61]. GWAS might indeed contribute to understand different ethnic (or sex related?) predisposition also in the recent SARS-CoV-2 pandemic, although disease susceptibility cannot be directly inferred from these data. Interestingly, however, by using available Italian exome studies, four SNPs were found to differ in Italian population compared with East Asians with the missense substitution p.Val160Met already associated with genomic rearrangement of TMPRSS2 as risk factor of prostate cancer. In addition and more interestingly, two other haplotypes are described, the first one proper of the European population and related to the up-regulation of *TMPRSS2* by androgens, and the second one characterized by three SNPs associated with higher *TMPRSS2* expression [46]. Along with this, it is attractive to observe that the *TMPRSS2:ERG* fusion gene is found in approximately 50% of prostate tumours of whites whereas is infrequent in black and Asian men [56].

Several other speculations may strengthen the role of sex into the expression of TMPRSS2. A first speculation derives from the androgens activity in vitro in the lung-derived cell line A549 to upregulate TMPRSS2 expression and to variably affect about 200 other transcripts including down-regulation of genes involved in cell respiration [62]. In the mouse, androgen receptor (AR) is expressed in type II pneumocytes and the bronchial epithelium where TMPRSS2 and ACE2 are expressed and it is regulated by androgen treatment in a positive loop. In humans, the AR is expressed in normal lungs as observed by immunohistochemistry with a signal which depends on the donors but it is independent of the sex [62]. The possible favourable role of androgen targeting in COVID-19, as already suggested [56], has been recently addressed in vivo in a observational study. Actually, prostate cancer patients receiving anti-androgenic therapy had a significantly lower risk of SARS-CoV-2 infection than alternative-treated patients and much lower when compared with patients affected by other tumor types [63]. However, the possible modulation of TMPRSS2 expression in lungs of treated individual has not been studied. As a fact, constitutive expression of TMPRSS2 and mRNA levels of the protein are equal in males and females although with high variability among the donors of the two sexes [56]. Further, post-menopausal women might be as susceptible as men because of the relative prevalence of male hormones and reduced levels of female hormones. The effects of oestrogens on TMPRSS2 expression and modulation are still largely unknown in normal conditions. Both oestrogen receptors (ERα and ERβ) are expressed on normal prostate cells but they would possibly mediate opposite effects at least in cancer, with up and down-regulation of *TMPRSS2*-*ERG*, respectively [64]. Thus, the central question about the degree of regulation of TMPRSS2 protein expression in the lung by androgen signalling still needs to be addressed.

### When SeXX matters in anti-viral responses

**Fig. 2:**
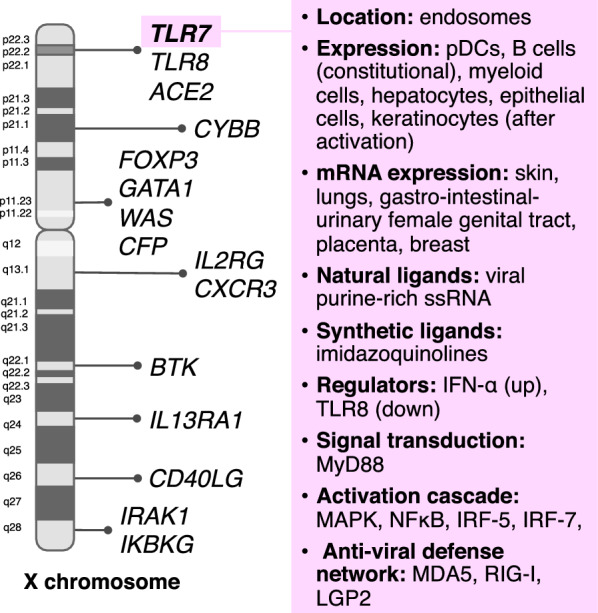
TLR7 within the X chromosome and its principal characteristics in the anti-viral response. The Toll-like Receptor 7 gene (*TLR7*) is located in the pseudo-autosomal region 1 of the X chromosome (p22.2). The principal characteristics of TLR7 are summarized on the right. ACE2: Angiotensin I Converting Enzyme 2; BTK: Bruton Tyrosine Kinase; CD40LG: CD40 Ligand; CFP: Complement Factor Properdin; CYBB: Cytochrome B-245 Beta Chain; CXCR3: C-X-C Motif Chemokine Receptor 3; GATA1: GATA Binding Protein 1; IKBKG: Inhibitor of Nuclear Factor Kappa B Kinase Regulatory Subunit Gamma; IL13RA1: Interleukin 13 Receptor Subunit Alpha 1; IL2RG:Interleukin 2 Receptor Subunit Gamma; IARK1: Interleukin 1 Receptor Associated Kinase 1; IRF Interferon regulatory factors; FOXP3: Forkhead Box P3; LGP2 laboratory of genetic and physiology 2; MAPK mitogen-activated protein kinase; MDA5 melanoma differentiation-associated protein 5; MyD88 myeloid differentiation primary response gene 88; NFκB nuclear factor kappa-light-chain-enhancer of activated B cells; pDCs plasmacytoid dendritic cells; RIG-I retinoic acid-inducible gene I; TLR8: Toll Like Receptor 8; WAS: Wiskott-Aldrich Syndrome

From *Drosophila melanogaster* on, the X chromosome harbours many of the genes encoding for innate signalling proteins thus giving a possible explanation for the observed sex-specific differences into defence mechanisms against viral, fungal or bacterial infections. On the other hand, *Sry* expression on the Y chromosome is related to the reduction of the immune response. The X chromosome is a giant chromosome containing about 3000 different genes and encoding for more than 800 miRNAs (Fig. 2). Genes of importance for a robust immune response are encoded on X such as CD40L, FoxP3, BTK, IL-2R gamma chain, WAS, properdin, and IKBKG, among the others [65]. As said, X inactivation has evolved to maintain equivalent gene expression into the two sexes, but a small subset of genes located in the non-recombining regions can escape this mechanism. Thus, their products are higher expressed in females and the pattern recognition receptors (PRRs) Toll-like receptor (TLR) 7 and TLR8 are examples of the XCI escape [66]. TLR7 is an endosomally located sensor detecting natural (viral) single-stranded ribonucleic acid or activated by synthetic ligands such as imidazoquinolines. It is constitutively expressed on plasmacytoid dendritic cells and B lymphocytes in addition to myeloid cells and non-immune cells such as hepatocytes, epithelial cells, and keratinocytes after induction. Both mRNA and protein are found in several tissues especially those lining with external (skin, lungs, gastro-intestinal and urinary tracts) and in female tissues (placenta, breast and genital tract) [67]. Up-regulation of TLR7 may results from viral infections such as influenza and this phenomenon is dependent on type I Interferons (IFN) which in turns is produced by TLR7 triggering. Upon ligand binding, TLR7 dimerizes and activates a well-known signalling pathway involving myeloid differentiation primary response gene 88 (MyD88), mitogen-activated protein kinase (MAPK) cascades, NF-κB activation, as well as IFN regulatory factor (IRF)-7 and IRF-5 activation via IL-1 receptor-associated kinases (IRAK)-1/2/4 and TNF receptor-associated factor-3/6 [68]. As a results, pro-inflammatory cytokines (TNF-α, IL-6, IL-1β, IL-12), and IFN-α are produced. Cooperation between TLR7 and other viral sensors collectively called RIG-1 like receptors (RLRs) (melanoma differentiation-associated protein 5 [MDA5], laboratory of genetic and physiology 2 [LGP2], retinoic acid inducible gene [RIG]-I), may operate to robustly respond to viral infections. Finally, TLR7 may stimulate B cells to enhanced antibody production. The activity of TLR8 is less studied as initially defined as inactive in experimental mouse models, but it is likely involved into regulatory mechanisms of expression of TLR7 itself [68].

Several lines of evidence related less susceptibility and severity and better outcomes of viral infections in women than men to increased TLR7 activity and/or expression. First, TLR7 expression is indeed augmented in women. Actually, biallelic expression of TLR7 has been found in primary B lymphocytes, monocytes, and plasmacytoid dendritic cells (pDCS) of women (and Klinefelter syndrome males) and this results in higher TLR7 protein expression in leukocyte populations of females [69]. Secondly, higher in vitro IFN-α production in females was noticed by several Authors after stimulation with synthetic TLR7 ligands. In a large cohort of healthy individuals, female peripheral blood lymphocytes produced much higher IFN-α levels than male after TLR7 stimulation with a striking significance (*p* < 0.0000001). No sex difference was noticed after TLR9 stimulation, in TNF-α production or numbers of responding pDCs. No in vitro effects of oestrogens were observed at that time [70]. A similar observation was also described by other groups [71]. Ten years later, the explanation was found into the sex different basal levels of IRF-5 in pDCs and positive correlation with the percentage of IFN-α–secreting pDCs in females [72]. An hormonal control of both TLR7 and IRF-5 might be involved. In humans, mRNA levels for IRF-5 correlate with oestrogen receptor 1 (ER1) expression suggesting a possible IRF-5 regulation by ER1 at transcriptional level [72]. TLR7 expression on immune cells is higher in females after puberty [73]. Also in mice, in vivo 17β-oestradiol directly targets pDCs and in vitro cytokine production by pDCs is promoted by TLR7 ligands by means of ERα expression [74]. However, it is difficult to disclose the relative role of X chromosome and hormones, at least in human beings. Interestingly, in XX females IFN-α production (and frequency of IFN-α producing pDCs) was always above than monosomy X (Turner’s syndrome) females and XY males. On the other hand, in transgender males (XX, highly testosterone treated) IFN-α production (and frequency of IFN-α producing pDCs) was significantly below than XX untreated females, whereas the same difference with a lower significance was also found when transgender females (XY, low testosterone) were compared to XY untreated males [73].

As the other side of the coin, this more robust immune response of females whatever is due, X chromosome, X numbers and/or sex hormones, while conditioning females as the stronger sex, also may contribute to more likely develop autoimmune diseases [75, 76].

### What Interleukin-sex are you? Protection by reduced inflammation

IL-6 has been claimed to be crucially involved into the dysregulated host immune response of critically ill COVID-19 patients (reviewed in [77]). Hypercytokinemia is the hallmark of the severe disease with notable increase in the levels of a plethora of cytokines and chemokines (IL-1β, IL-2, IL-6, IL-17, IFN-ɣ, IL-8, IL-18, TNF, and CCL2 among the others) with IL-10 attempting to dampen the exaggerated immune activation [78]. As a consequence, patients not only develop a severe pneumonia but also exhibit a sort of macrophage activation syndrome/cytokine release syndrome (CRS) with deranged liver function tests, diffuse endothelial damage and coagulopathy, mirrored by circulating high D-dimer, hyperferritinaemia and high C-reactive protein evolving towards a multi-organ failure [79]. It is known since years that persistent elevation of plasma levels of IL-1β and IL-6 are predictors of poor outcome in ARDS [80]. Accordingly, IL-6 levels in patients requiring intensive care after SARS-CoV-2 infection increase over time and are higher in non-survivors than survivors [26]. IL-6 indeed plays a crucial role into the CRS and IL-6 levels together with C-reactive protein and SaO_2_/FiO_2_ ratio would be predictors of rapid clinical deterioration in COVID-19 patients [81]. As a fact, tocilizumab is likely to be an effective drug for patients with severe COVID-19 [82] and anti-IL-6R strategy has been related with the restoration of NK cells killing machinery, profoundly affected in severely ill patients and also observed in macrophage activation syndrome [83].

In normal individuals, IL-6 is broadly expressed being bone marrow and lymphoid tissues, fat, lungs, bladder and urinary tract the most enriched tissues [67] and prevalent in women. Despite this, a buffered inflammatory response is claimed as responsible of the longer lifespan, better health and better outcomes after severe injuries of females. After traumatic injury or haemorrhagic shock males would exhibit persistently elevated IL-6 levels which have been associated with a higher rate of multiple organ failure [84] even if these data have been recently debated [85]. In experimental models, stress gene expression levels and IL-6 are higher in male than female mice after LPS challenge [86]. In humans, the stimulation of whole blood with TLR-4, 7/8 and 2 ligands always produced lower IL-6 levels in women than in men [87]. With an elegant approach combining ATAC-seq, RNA-seq, and flow cytometry analysis of the peripheral blood mononuclear cells from 172 healthy adults (22–93 years of age) of both sexes revealed that monocyte-specific loci were 15-fold increased in men although the cell numbers were comparable. Along with this, IL18BP and IL-6 levels, even if increasing with age, were always higher in men than females [88]. It is difficult to dissect the relative importance of steroid hormones and X-chromosomes in this immune disparity.

## Conclusions

Emerging evidence shows that COVID-19 is a gender-biased disease influenced by a myriad of variables ranging from biological to social factors. Although it is still premature to draw firm conclusions about gender and SARS-CoV-2 infection, gender equality must be a priority in the fight against COVID-19.

## Data Availability

Not applicable

## Abbreviations

ACE2: Angiotensin-converting enzyme 2
ANG: Angiotensin
AR: Androgen receptor
ARDS: Acute respiratory distress syndrome
AT1R: Angiotensin II type 1 receptor
AT2R: Angiotensin II type 2 receptor
CAP: Community acquired pneumonia
CFR: Case fatality rate
COVID-19: Coronavirus disease 2019
CRS: Cytokine release syndrome
ER: Oestrogen receptor
GWAS: Genome-wide association studies
IFN: Interferon
IL-1β: Interleukin-1β
IL-6: Interleukin-6
IL-10: Interleukin-10
MERS: Middle East respiratory syndrome
PAR: Pseudoautosomal region
RAS: Renin angiotensin system
SARS: Severe acute respiratory syndrome
SNP: Single-nucleotide polymorphism
TLR7: Toll-like receptor 7
TLR8: Toll-like receptor 8
TMPRSS2: Type 2 transmembrane serine protease
TNF-α: Tumor necrosis factor-α
WT: Wild type
XCI: X-chromosome inactivation

## References

[1] Ingersoll MA (2017). Sex differences shape the response to infectious diseases. PLoS Pathog.

[2] World Health Organization. WHO Website. Coronavirus disease (COVID-2019) situation reports. Situation Report 89. https://www.who.int/emergencies/diseases/novel-coronavirus-2019/situation-reports. Accessed 25 Apr 2020.

[3] Gudbjartsson DF, Helgason A, Jonsson H (2020). Spread of SARS-CoV-2 in the Icelandic population. N Engl J Med..

[4] Global Health 5050. Global Health 5050 Website. Sex, gender and COVID-19. https://globalhealth5050.org/covid19. Accessed 7 June 2020.

[5] Bhopal R (2020). Covid-19 worldwide: we need precise data by age group and sex urgently. BMJ.

[6] Jin JM, Bai P, He W (2020). Gender differences in patients with COVID-19: focus on severity and mortality. Front Public Health..

[7] Bischof E, Wolfe J, Klein SL (2020). Clinical trials for COVID-19 should include sex as a variable. J Clin Invest..

[8] World Health Organization. WHO Website. Coronavirus disease (COVID-2019) situation reports. Situation Report 135. https://www.who.int/emergencies/diseases/novel-coronavirus-2019/situation-reports. Accessed 7 June 2020.

[9] Istituto Superiore di Sanità. The COVID-19 Task force of the Department of Infectious Diseases and the IT Service Istituto Superiore di Sanità. Infographic of the COVID-19 Italian Integrated Surveillance Data as of 3rd June 2020. https://www.epicentro.iss.it/en/coronavirus/sars-cov-2-integrated-surveillance-data. Accessed 7 June 2020.

[10] Istituto Superiore di Sanità. Italian SARS-CoV-2 Surveillance Group. Characteristics of SARS-CoV-2 patients dying in Italy—report based on available data on June 4th, 2020 https://www.epicentro.iss.it/en/coronavirus/sars-cov-2-analysis-of-deaths. Accessed 7 June 2020.

[11] Istituto Nazionale per l’Assicurazione contro gli Infortuni sul Lavoro. Consulenza statistico attuariale. 30th April 2020. https://www.inail.it/cs/internet/comunicazione/news-ed-eventi/news/news-denunce-contagi-covid-aprile-2020.html&tipo=news. Accessed 20 May 2020.

[12] Grasselli G, Zangrillo A, Zanella A (2020). Baseline characteristics and outcomes of 1591 patients infected with SARS-CoV-2 admitted to ICUs of the Lombardy Region, Italy. JAMA.

[13] Lagi F, Piccica M, Graziani L (2020). Early experience of an infectious and tropical diseases unit during the coronavirus disease (COVID-19) pandemic, Florence, Italy, February to March 2020. Euro Surveill.

[14] Guan WJ, Ni ZY, Hu Y (2020). Clinical characteristics of coronavirus disease 2019 in China. N Engl J Med.

[15] Onder G, Rezza G, Brusaferro S (2020). Case-fatality rate and characteristics of patients dying in relation to COVID-19 in Italy. JAMA.

[16] Wenham C, Smith J, Morgan R, Gender and COVID-19 Working Group (2020). COVID-19: the gendered impacts of the outbreak. Lancet.

[17] Gustafson PE (1998). Gender differences in risk perception: theoretical and methodological perspectives. Risk Anal.

[18] Moran KR, Del Valle SY (2016). A meta-analysis of the association between gender and protective behaviors in response to respiratory epidemics and pandemics. PLoS ONE.

[19] Falagas ME, Mourtzoukou EG, Vardakas KZ (2007). Sex differences in the incidence and severity of respiratory tract infections. Respir Med.

[20] Reade MC, Yende S, D’Angelo G, Genetic and Inflammatory Markers of Sepsis Investigators (2009). Differences in immune response may explain lower survival among older men with pneumonia. Crit Care Med..

[21] Karlberg J, Chong DS, Lai WY (2004). Do men have a higher case fatality rate of severe acute respiratory syndrome than women do?. Am J Epidemiol.

[22] Re Goggins W (2004). Do men have a higher case fatality rate of severe acute respiratory syndrome than women do?. Am J Epidemiol.

[23] Aly M, Elrobh M, Alzayer M (2017). Occurrence of the middle east respiratory syndrome coronavirus (MERS-CoV) across the Gulf Corporation Council countries: four years update. PLoS ONE.

[24] Jansen A, Chiew M, Konings F, Lee CK, Ailan L, on behalf the World Health Organization Regional Office for the Western Pacific MERS Event Management Team (2015). Sex matters—a preliminary analysis of Middle East respiratory syndrome in the Republic of Korea, 2015. Western Pac Surveill Response J..

[25] Chen X, Chughtai AA, Dyda A, MacIntyre CR (2017). Comparative epidemiology of Middle East respiratory syndrome coronavirus (MERS-CoV) in Saudi Arabia and South Korea. Emerg Microbes Infect..

[26] Tay MZ, Poh CM, Rénia L (2020). The trinity of COVID-19: immunity, inflammation and intervention. Nat Rev Immunol.

[27] Yang SH, Gao CY, Li L (2018). The molecular basis of immune regulation in autoimmunity. Clin Sci (Lond)..

[28] Li F, Li W, Farzan M, Harrison SC (2005). Structure of SARS coronavirus spike receptor-binding domain complexed with receptor. Science.

[29] Hoffmann M, Kleine-Weber H, Schroeder S (2020). SARS-CoV-2 cell entry depends on ACE2 and TMPRSS2 and is blocked by a clinically proven protease inhibitor. Cell.

[30] Santos RAS, Oudit GY, Verano-Braga T (2019). The renin-angiotensin system: going beyond the classical paradigms. Am J Physiol Heart Circ Physiol..

[31] Gheblawi M, Wang K, Viveiros A (2020). Angiotensin-converting enzyme 2: SARS-CoV-2 receptor and regulator of the renin-angiotensin system: celebrating the 20th anniversary of the discovery of ACE2. Circ Res.

[32] Rompe F, Unger T, Steckelings UM (2010). The angiotensin AT2 receptor in inflammation. Drug News Perspect..

[33] Santos RAS, Sampaio WO, Alzamora AC (2018). The ACE2/angiotensin-(1–7)/MAS axis of the renin-angiotensin system: focus on angiotensin-(1–7). Physiol Rev.

[34] Nicholls J, Peiris M (2005). Good ACE, bad ACE do battle in lung injury. SARS. Nat Med..

[35] Bao L, Deng W, Huang B (2020). The pathogenicity of SARS-CoV-2 in hACE2 transgenic mice. Nature.

[36] Bertram S, Heurich A, Lavender H (2012). Influenza and SARS-coronavirus activating proteases TMPRSS2 and HAT are expressed at multiple sites in human respiratory and gastrointestinal tracts. PLoS ONE.

[37] Fang F, Liu GC, Zhou X (2013). Loss of ACE2 exacerbates murine renal ischemia-reperfusion injury. PLoS ONE.

[38] Gu H, Xie Z, Li T (2016). Angiotensin-converting enzyme 2 inhibits lung injury induced by respiratory syncytial virus. Sci Rep..

[39] Khan A, Benthin C, Zeno B (2017). A pilot clinical trial of recombinant human angiotensin-converting enzyme 2 in acute respiratory distress syndrome. Crit Care.

[40] Jia H (2016). Pulmonary Angiotensin-Converting Enzyme 2 (ACE2) and inflammatory lung disease. Shock.

[41] Kuba K, Imai Y, Rao S (2005). A crucial role of angiotensin converting enzyme 2 (ACE2) in SARS coronavirus-induced lung injury. Nat Med.

[42] Glowacka I, Bertram S, Herzog P (2010). Differential downregulation of ACE2 by the spike proteins of severe acute respiratory syndrome coronavirus and human coronavirus NL63. J Virol.

[43] Mangs AH, Morris BJ (2007). The human pseudoautosomal region (PAR): origin, function and future. Curr Genomics.

[44] Zhao Y, Zhao Z, Wang Y, et al. Single-cell RNA expression profiling of ACE2, the receptor of SARS-CoV-2. [preprint] 10.1101/2020.01.26.919985. 10.1164/rccm.202001-0179LE PMC7462411 32663409

[45] Cao Y, Li L, Feng Z, Wan S (2020). Comparative genetic analysis of the novel coronavirus (2019-nCoV/SARS-CoV-2) receptor ACE2 in different populations. Cell Discov..

[46] Asselta R, Paraboschi EM, Mantovani A, Duga S (2020). ACE2 and TMPRSS2 variants and expression as candidates to sex and country differences in COVID-19 severity in Italy. Aging (Albany NY).

[47] Stawiski EW, Diwanji D, Suryamohan K, et al. Human ACE2 receptor polymorphisms predict SARS-CoV-2 susceptibility. [preprint] 10.1101/2020.04.07.024752.

[48] Xie X, Chen J, Wang X (2006). Age- and gender-related difference of ACE2 expression in rat lung. Life Sci.

[49] Chen K, Bi J, Su Y (2016). Sex-specific changes in renal angiotensin-converting enzyme and angiotensin-converting enzyme 2 gene expression and enzyme activity at birth and over the first year of life. Reprod Sci..

[50] Fernández-Atucha A, Izagirre A, Fraile-Bermúdez AB (2017). Sex differences in the aging pattern of renin-angiotensin system serum peptidases. Biol Sex Differ..

[51] Hilliard LM, Sampson AK, Brown RD, Denton KM (2013). The “his and hers” of the renin-angiotensin system. Curr Hypertens Rep.

[52] Komukai K, Mochizuki S, Yoshimura M (2010). Gender and the renin-angiotensin-aldosterone system. Fundam Clin Pharmacol.

[53] Brosnihan KB, Hodgin JB, Smithies O (2008). Tissue-specific regulation of ACE/ACE2 and AT1/AT2 receptor gene expression by oestrogen in apolipoprotein E/oestrogen receptor-alpha knock-out mice. Exp Physiol.

[54] Neves LA, Stovall K, Joyner J (2008). ACE2 and ANG-(1–7) in the rat uterus during early and late gestation. Am J Physiol Regul Integr Comp Physiol.

[55] Paoloni-Giacobino A, Chen H, Peitsch MC (1997). Cloning of the TMPRSS2 gene, which encodes a novel serine protease with transmembrane, LDLRA, and SRCR domains and maps to 21q22.3. Genomics..

[56] Stopsack KH, Mucci LA, Antonarakis ES (2020). TMPRSS2 and COVID-19: serendipity or opportunity for intervention?. Cancer Discov.

[57] Lin B, Ferguson C, White JT (1999). Prostate-localized and androgen-regulated expression of the membrane-bound serine protease TMPRSS2. Cancer Res.

[58] Shen LW, Mao HJ, Wu YL (2017). TMPRSS2: a potential target for treatment of influenza virus and coronavirus infections. Biochimie.

[59] Eshima N, Tokumaru O, Hara S (2011). Sex- and age-related differences in morbidity rates of 2009 pandemic influenza A H1N1 virus of swine origin in Japan. PLoS ONE.

[60] Morgan R, Klein SL (2019). The intersection of sex and gender in the treatment of influenza. Curr Opin Virol..

[61] Cheng Z, Zhou J, To KK (2015). Identification of TMPRSS2 as a susceptibility gene for severe 2009 pandemic A(H1N1) Influenza and A(H7N9) Influenza. J Infect Dis.

[62] Mikkonen L, Pihlajamaa P, Sahu B (2010). Androgen receptor and androgen-dependent gene expression in lung. Mol Cell Endocrinol.

[63] Montopoli M, Zumerle S, Vettor R (2020). Androgen-deprivation therapies for prostate cancer and risk of infection by SARS-CoV-2: a population-based study (n = 4532). Ann Oncol.

[64] Setlur SR, Mertz KD, Hoshida Y (2008). Estrogen-dependent signaling in a molecularly distinct subclass of aggressive prostate cancer. J Natl Cancer Inst.

[65] Libert C, Dejager L, Pinheiro I (2010). The X chromosome in immune functions: when a chromosome makes the difference. Nat Rev Immunol.

[66] Ghosh S, Klein RS (2017). Sex drives dimorphic immune responses to viral infections. J Immunol..

[67] The Human Protein Atlas. The Human Protein Atlas Website. https://www.proteinatlas.org. Accessed 20 May 2020.

[68] Petes C, Odoardi N, Gee K (2017). The toll for trafficking: toll-like receptor 7 delivery to the endosome. Front Immunol..

[69] Souyris M, Cenac C, Azar P (2018). TLR7 escapes X chromosome inactivation in immune cells. Sci Immunol..

[70] Berghöfer B, Frommer T, Haley G (2006). TLR7 ligands induce higher IFN-alpha production in females. J Immunol..

[71] Torcia MG, Nencioni L, Clemente AM (2012). Sex differences in the response to viral infections: TLR8 and TLR9 ligand stimulation induce higher IL10 production in males. PLoS ONE.

[72] Griesbeck M, Ziegler S, Laffont S (2015). Sex differences in plasmacytoid dendritic cell levels of IRF5 drive higher IFN-α production in women. J Immunol..

[73] Webb K, Peckham H, Radziszewska A (2019). Sex and pubertal differences in the type 1 interferon pathway associate with both X chromosome number and serum sex hormone concentration. Front Immunol..

[74] Seillet C, Laffont S, Trémollières F (2012). The TLR-mediated response of plasmacytoid dendritic cells is positively regulated by estradiol in vivo through cell-intrinsic estrogen receptor α signaling. Blood.

[75] Rubtsova K, Marrack P, Rubtsov AV (2015). Sexual dimorphism in autoimmunity. J Clin Invest..

[76] Ngo ST, Steyn FJ, McCombe PA (2014). Gender differences in autoimmune disease. Front Neuroendocrinol.

[77] Coomes EA, Haghbayan H. Interleukin-6 in COVID-19: a systematic review and meta-analysis. [preprint] 10.1101/2020.03.30.20048058. 10.1002/rmv.2141 PMC7460877 32845568

[78] McGonagle D, Sharif K, O’Regan A, Bridgewood C (2020). The role of cytokines including interleukin-6 in COVID-19 induced pneumonia and macrophage activation syndrome-like disease. Autoimmun Rev.

[79] Zhou F, Yu T, Du R (2020). Clinical course and risk factors for mortality of adult inpatients with COVID-19 in Wuhan, China: a retrospective cohort study. Lancet.

[80] Meduri GU, Headley S, Kohler G (1995). Persistent elevation of inflammatory cytokines predicts a poor outcome in ARDS. Plasma IL-1 beta and IL-6 levels are consistent and efficient predictors of outcome over time. Chest.

[81] Vultaggio A, Vivarelli E, Virgili G (2020). Prompt Predicting of Early Clinical Deterioration of Moderate-to-Severe COVID-19 Patients: Usefulness of a Combined Score Using IL-6 in a Preliminary Study. J Allergy Clin Immunol Pract..

[82] Luo P, Liu Y, Qiu L (2020). Tocilizumab treatment in COVID-19: a single center experience. J Med Virol..

[83] Mazzoni A, Salvati L, Maggi L (2020). Impaired immune cell cytotoxicity in severe COVID-19 is IL-6 dependent. J Clin Invest.

[84] Sperry JL, Friese RS, Frankel HL (2008). Male gender is associated with excessive IL-6 expression following severe injury. J Trauma.

[85] Lopez MC, Efron PA, Ozrazgat-Baslanti T (2016). Sex-based differences in the genomic response, innate immunity, organ dysfunction, and clinical outcomes after severe blunt traumatic injury and hemorrhagic shock. J Trauma Acute Care Surg..

[86] Everhardt Queen A, Moerdyk-Schauwecker M, McKee LM (2016). Differential expression of inflammatory cytokines and stress genes in male and female mice in response to a lipopolysaccharide challenge. PLoS ONE.

[87] Lefèvre N, Corazza F, Valsamis J (2019). The number of X chromosomes influences inflammatory cytokine production following toll-like receptor stimulation. Front Immunol.

[88] Márquez EJ, Chung CH, Marches R, Rossi RJ, Nehar-Belaid D, Eroglu A, Mellert DJ, Kuchel GA, Banchereau J, Ucar D (2020). Sexual-dimorphism in human immune system aging. Nat Commun..

